# Static Drainage H_2_/O_2_ PEM Fuel Cells Enabled by Water Transport Plates: Pore‐Scale Design and Transport Mechanisms

**DOI:** 10.1002/advs.77662

**Published:** 2026-09-13

**Authors:** Mengdi Guo, Diankai Qiu, Dong Zhu, Linfa Peng

**Affiliations:** ^1^ Shanghai Key Laboratory of Digital Manufacture for Thin‐walled Structures Shanghai Jiao Tong University Shanghai People's Republic of China; ^2^ State Key Laboratory of Mechanical System and Vibration Shanghai Jiao Tong University Shanghai People's Republic of China; ^3^ National Engineering Research Center of Automotive Power and Intelligent Control Shanghai Jiao Tong University Shanghai People's Republic of China

**Keywords:** dead‐ended anode and cathode, pore network model, proton exchange membrane fuel cell, water management, water transport plate

## Abstract

As the suitability of proton exchange membrane fuel cells (PEMFCs) for underwater and space applications gains broader recognition, dead‐ended operation has attracted increasing attention. However, water management challenges are exacerbated in PEMFCs without high‐rate purge flows. Flooding frequently occurs, and thus hinders long‐term stable operation. In this study, water transport plates (WTPs) were specifically designed to concurrently satisfy the dual requirements of passive water removal and gas tightness for dead‐ended cathode operation. A pore‐network model was established to investigate the capillary behavior mechanism within the porous structure, predict the water flux and bubble pressure of the WTPs, and further optimize the design of key parameters including average pore diameter. The network topology is reconstructed from X‐CT images, and pore‐throat shape corrections together with throat tortuosity are incorporated. By taking water flux (≥27.9 µL·cm^−2^·min^−1^) and bubble pressure (≥40 kPa) as evaluation indicators, the average pore diameter range of 1.2 ∼ 4.2 µm was selected. The optimized WTP was fabricated and assembled into a fuel cell stack. The stack operated stably for over 30 h with 1.92% performance decrease under dead‐ended cathode operation at 1A·cm^−2^. These findings offer insights for the design and performance enhancement of dead‐end PEMFCs with static drainage mode.

## Graphical abstract

The WTP is a key component of static drainage PEMFCs, enabling cathode water removal and gas sealing through capillary forces. This work investigates the mechanisms governing WTP drainage and sealing performance and develops predictive approaches for WTP design optimization. The optimized WTP enables prolonged stable operation of PEMFCs under dead‐end cathode conditions.

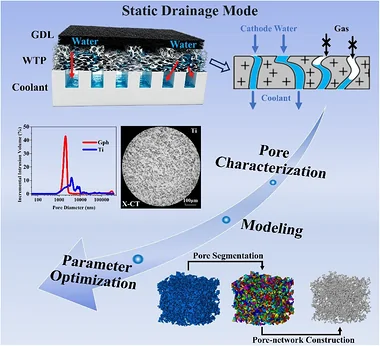

## 1 Introduction

Proton exchange membrane fuel cells (PEMFCs) generate electricity through electrochemical reactions between hydrogen and oxygen [1, 2, 3, 4, 5]. Owing to high efficiency, low operating temperature, quiet operation, and zero local emissions, PEMFCs have been extensively investigated and deployed in various fields, including transportation, stationary power plants, submarines, and spacecraft [6, 7, 8, 9, 10]. In underwater and space environments, where air supply is unavailable and system volume is strictly limited, fuel cell systems require higher reactant utilization, reduced system volume, and simplified auxiliary components [11, 12, 13]. As an alternative to air, high‐purity oxygen is commonly used as the oxidant. Currently, fuel cells used in these environments typically operate in a dead‐end anode and cathode (DEAC) mode, minimizing or eliminating oxygen discharge to reduce waste of reactant gases and simplifying the system [14, 15]. However, closing the cathode gas outlet makes timely water removal difficult, resulting in severe water accumulation and increased flooding risk, and presenting significant challenges for water management [16, 17, 18, 19]. Therefore, more efficient water management strategies are urgently needed to address these issues.

Currently, there are several component‐assisted methods to mitigate flooding in dead‐end cathode fuel cells, including purging, recirculation, and the application of disturbances [20, 21, 22]. Tan‐Thich et al. [23] employed cathode purging and investigated the impact of different purging durations and intervals on fuel utilization and cell performance, proposing an optimal cathode purging strategy. Alizadeh et al. [24] used liquid separators to separate liquid water, then reintroduced the gas into the stacks. Fan et al. [25] used a buffer tank to collect all the gas from the purging phase, separate the expelled liquid water, significantly reducing the performance degradation caused by flooding. Choi et al. [26] employed a mechanical piston device to apply pulse disturbances to the cathode gas. Pei et al. [27] also added a buffer tank at the cathode gas outlet of the fuel cell and controlled the cathode gas pressure fluctuations by adjusting the electromagnetic valve. However, the active drainage mechanisms mentioned above mostly rely on gas flow to remove liquid water. They require additional system components, such as circulation pump, buffer tank and water separator, which introduce parasitic power losses and increase system complexity. In underwater and space environments, this results in space wastage and reduces overall energy efficiency.

**FIGURE 1:**
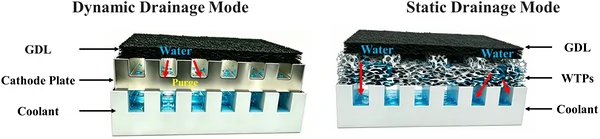
Dynamic and static drainage fuel cells.

Recently, static drainage fuel cells have attracted growing attention [28, 29]. In this concept, as illustrated in Figure 1, a water transport plate (WTP) used at cathode is fabricated from a porous, hydrophilic material, positioned between the cooling water channel and the gas channel. Liquid water is removed directly from cathode side into the coolant, eliminating the need for gas purging. During operation, once the generated water reaches the gas diffusion layer (GDL)/gas channel (GC) interface, capillary action spontaneously wicks it into the porous matrix. Under the pressure difference between the oxygen and coolant channels, water is passively discharged into the coolant channels, thereby realizing passive in‐cell water removal. Meanwhile, after water is imbibed into the capillaries of the porous plate, the capillary force established at the liquid–gas interface balances the pressure difference and prevents gas leakage. Because static drainage PEMFCs require no high‐velocity purge gas, they substantially simplify the system and improve system efficiency, making them a particularly attractive option for dead‐end mode.

Flow field design also plays a critical role in determining the water management capability of fuel cells. Numerous studies have focused on optimizing and innovating flow field architectures in flow‐through fuel cells to enhance in‐plane water removal and improve reactant distribution uniformity [30, 31, 32, 33, 34, 35]. Some researchers [30, 31, 32] have optimized the geometries and operating parameters of conventional flow fields, while others [33, 34, 35] have proposed biomimetic configurations inspired by leaf‐vein and honeycomb structures. However, under the dead‐end cathode mode, flow field design places greater emphasis on through‐plane water transport. At present, relatively few studies have focused on optimizing flow field structures specifically for static drainage fuel cells, as most research has instead concentrated on the application and performance of WTPs in fuel cells [36, 37, 38, 39, 40, 41].

Guo et al. [36, 37] treated porous graphite materials with hydrophilic modifications and applied them as WTPs in dead‐end fuel cells. They measured parameters such as bubble pressure and permeability to assess the performance of the WTPs, and found that it effectively reduced the rate of liquid water accumulation within the cell. The WTP permeability was reported as 6.53 × 10^−16^ m^2^. Wang et al. [38, 39] employed in situ measurements to further quantify the water transport capabilities of the WTPs during cell operation. Under the dead‐end cathode mode, compared with the solid plate (SP), the purge cycle was extended from 30 s to more than 300 s. Cheng et al. [40] used sintered porous titanium as the WTPs material. Additionally, they conducted experiments to test the water transport ability of porous titanium, and the liquid flux was 148.8 µL·cm^−2^·min^−1^. The PEMFC operating in dead‐end mode at 1 A·cm^−2^ was maintained for 632 s, confirming that it effectively facilitated both humidification and water drainage. Lu et al. [41] proposed a novel fuel cell structure that incorporated capillary arrays designed to spontaneously drain water, mimicking the microstructure of respiratory cilia, which enhanced the fuel cell performance. These studies demonstrate the potential of WTPs for water management under dead‐end operation. Nevertheless, flooding still occurs in fuel cells, and periodic purging remains necessary to restore the cell voltage. In addition, WTPs also fail to achieve stable sealing sometimes. This is because WTPs contain a large number of non‐uniform microscopic pores, the size of which has opposing effects on water transport capacity and bubble pressure. A higher bubble pressure generally corresponds to smaller pore sizes and reduced water transport capability. The relationship between the drainage capacity, bubble pressure of the WTPs and its material parameters remains unclear, which hampers the ability to predict whether the WTPs will meet the operational requirements during the material selection phase. Hence, further optimization of WTPs design is still required, with both drainage capacity and bubble pressure serving as key performance indicators. It is essential to clearly understand how the parameters of porous materials influence both drainage capability and bubble pressure.

In this work, a design methodology for WTPs with rapid passive water transport and stable gas sealing capability was developed. A predictive model for the water‐gas transport performance of WTPs was introduced, along with parameter design and experimental verification. First, the WTPs were characterized using X‐CT scanning, mercury intrusion testing, and other techniques. Next, considering pore‐scale parameters such as pore shape and pore size distribution, a pore network model was built to predict the water flux and bubble pressure of the WTPs. Based on these predictions, the parameters of WTPs were designed, and optimized WTPs were fabricated and integrated into the fuel cell. Then, the static drainage structure of a hydrogen‐oxygen fuel cell was designed. The design incorporated a combination of porous and solid plates, producing a small‐thickness bipolar plate with high water flux and sealing performance. Finally, a two‐cell hydrogen–oxygen fuel cell stack was evaluated under dead‐ended cathode operation, achieving stable operation for 30 h.

## 2 Experimental Section

### 2.1 Characterization of Porous Bipolar Plates

In the flow field region of static drainage fuel cell bipolar plate, the traditional solid plate has been replaced by the WTPs. Driven by the pressure difference, the water at the cathode can be directly discharged into the coolant through the capillaries on the WTPs. The WTPs are mainly made of conductive hydrophilic porous materials, such as graphite, titanium, stainless steel. The porous plate sintered from powder has complex internal pore shapes and varying pore sizes. To investigate the influence of pore‐scale structural parameters on water transport capacity and bubble pressure, it is necessary to observe the internal pore structure of the materials and quantify the pore parameters. In this study, porous graphite and porous titanium were selected for parameter characterization.

The porous plates were ultrasonically cleaned by immersion in anhydrous ethanol to remove surface oils and other contaminants, then air‐dried to evaporate the solvent. The treated plates were subsequently subjected to structural characterization, contact‐angle measurements, and pore‐scale parameter determination. Pore structure parameter measurement and morphology observation were also carried out on the porous titanium selected via pore parameter optimization.

#### 2.1.1 Pore‐Scale Parameter Characterization

**FIGURE 2:**
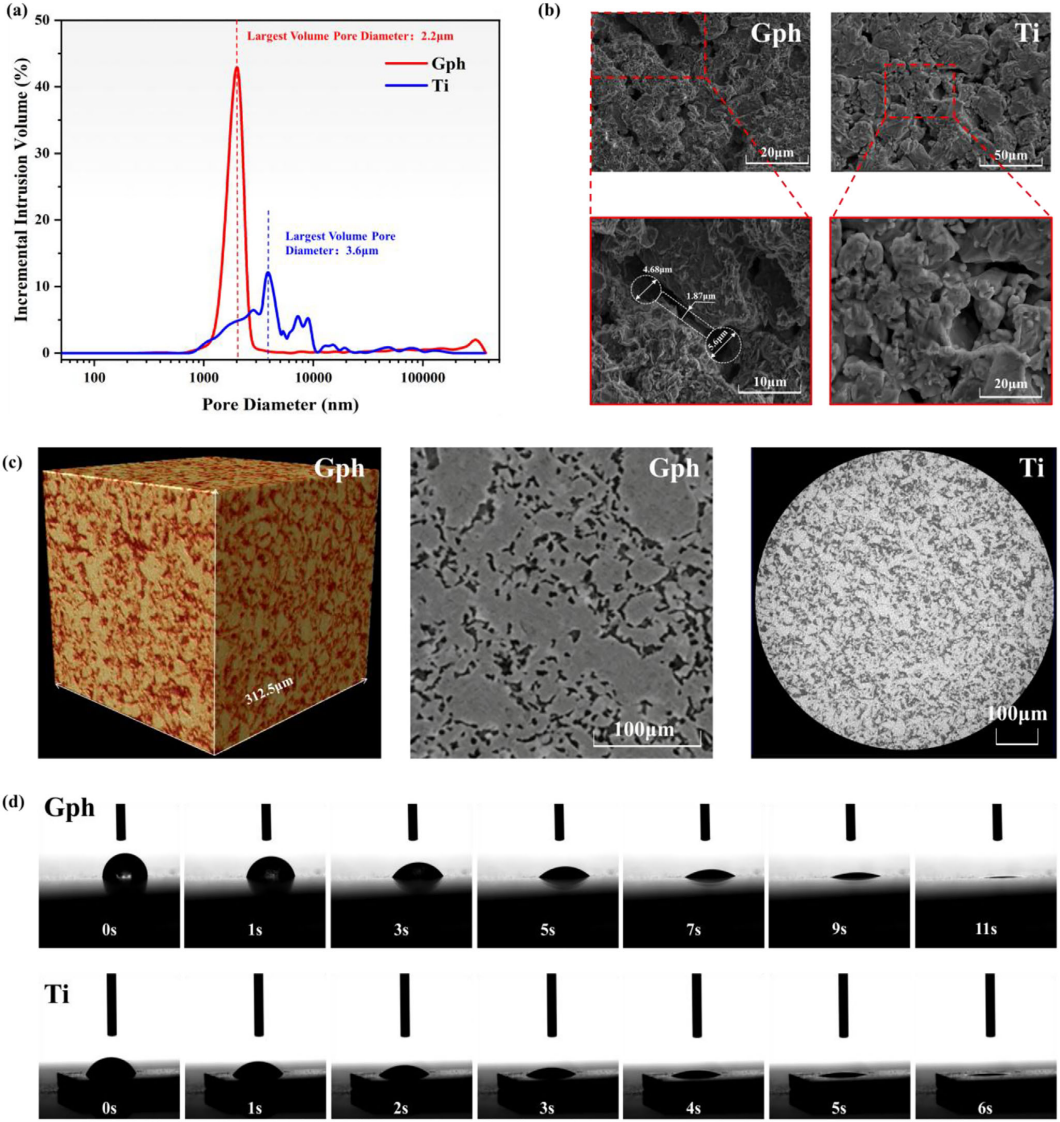
Characterization of porous bipolar plates: (a) Pore size distribution; (b) SEM image; (c) x‐ray image; (d) Contact angle test of porous plates.

To elucidate the influence of pore‐scale parameters on two‐phase transport, mercury intrusion porosimetry (MIP) was employed to determine the pore‐size distribution and porosity of the porous plate. As shown in Figure 2a, the material exhibits a distribution spanning nanometer to micrometer scales. The pore‐size distribution of porous graphite approximately follows a normal distribution, concentrated in the range of 1 to 3 µm. The pore size corresponding to the maximum volume fraction is approximately 2.2 µm. Furthermore, the volume fraction of interconnected pores in the graphite, measured by MIP, is 13.8%. For the porous titanium, the pore size distribution is more dispersed, the pore diameter with the largest volume fraction is 3.6 µm, and the porosity is 21.9%. In terms of pore size distribution, graphite exhibits a more uniform and concentrated distribution, whereas titanium shows a more dispersed and non‐uniform distribution.

#### 2.1.2 Microstructural characterization

After cutting the porous plates, the cross sections were examined for surface morphology. Scanning electron microscopy (SEM; RISE‐MAGNA) revealed the surface structures shown in Figure 2b. Micrometer‐scale pores are present within the porous plates and are interconnected by narrow throats; the pore shapes are complex. However, quantitative extraction of three‐dimensional pore‐shape descriptors is still required to enable construction of a pore‐network model.

Furthermore, the internal microstructure was imaged using x‐ray computed tomography (X‐CT; Xradia 520 Versa). Figure 2c shows the 3D X‐CT reconstruction. The isotropic voxel size was 0.3125 µm for porous graphite and 0.7 µm for porous titanium, with 1000 pixels along each of the x, y, and z axes.

#### 2.1.3 Contact Angle Measurement

To characterize the wettability of the porous plate surface, the sessile‐drop method was employed. A 1 µL water droplet was deposited on the plate, and the three‐phase contact angle was calculated using a drop‐shape analyzer (KRUSS). Because the porous plate gradually imbibes the droplet, as shown in Figure 2d, the contact angle exhibits a gradual decrease. Titanium is more hydrophilic, with a lower initial contact angle and faster liquid droplet absorption.

#### 2.1.4 Permeated Water Flux Measurement

**FIGURE 3:**
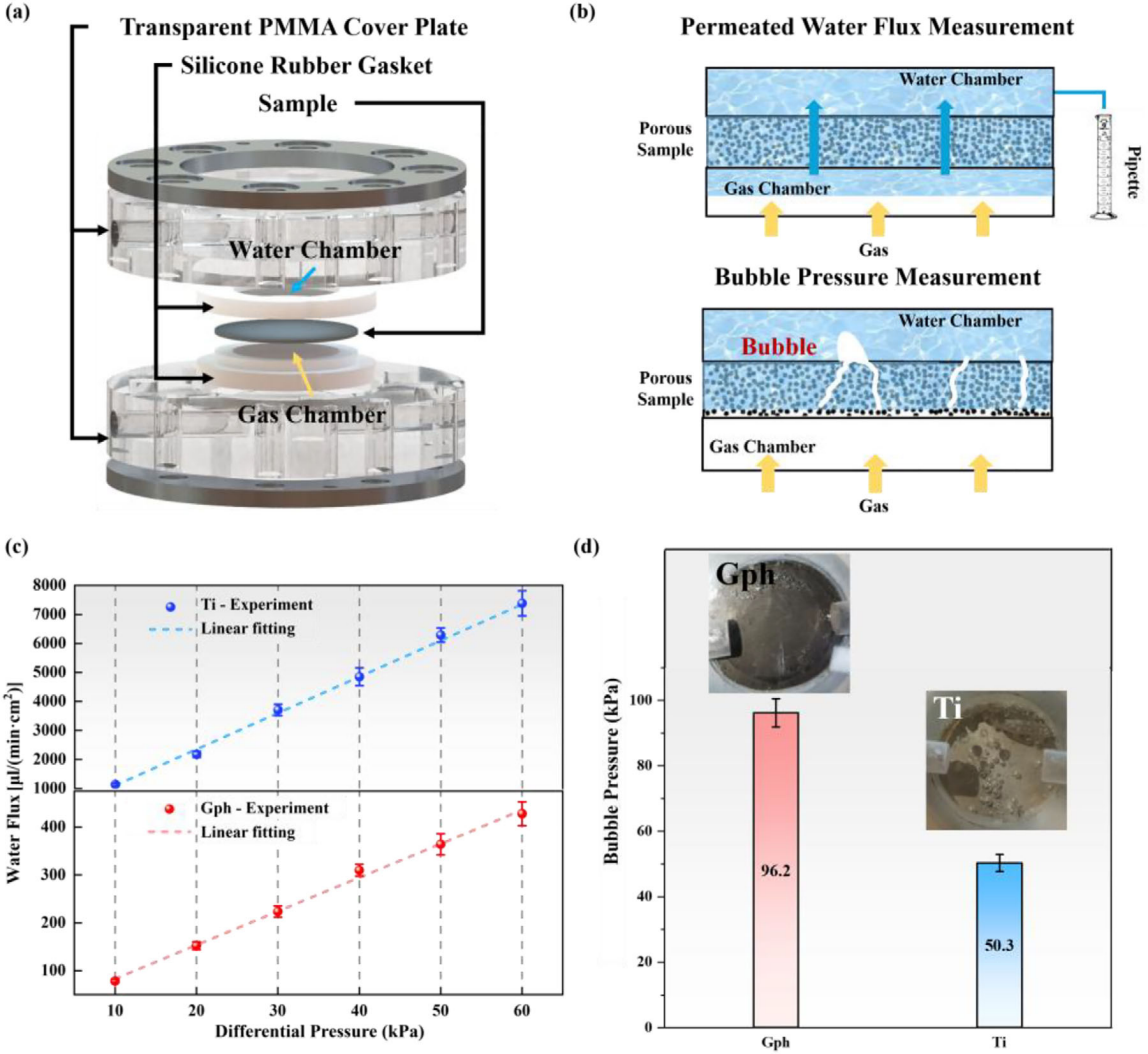
(a) The custom fixture; (b) Water flux and bubble pressure measurement; (c) Water flux under various pressure differentials; (d) Bubble pressure of samples.

As the key static drainage fuel cell component, the drainage capability of WTPs, quantified by the water flux, should be measured. As shown in Figure 3a, a custom fixture was designed to measure both water flux and bubble pressure. The sample is clamped between a water chamber and a gas chamber, and the water chamber is connected to a graduated pipette for volumetric readout. A transparent PMMA (acrylic) cover plate allows direct visualization of water penetration and bubble formation, and a silicone rubber gasket provides sealing. A compressive load of 2 MPa was applied to seal the edges of the graphite sample.

For water flux testing (Figure 3b), water is first introduced into the water chamber and its pressure is raised so that water back‐fills into the gas chamber until the latter is completely filled with water. Then, according to Cheng et al. [40], the operational ΔP between the gas chamber and liquid chamber is set from 10 to 60 kPa. preventing gas leakage, and the water flux is recorded. The corresponding water flux was recorded under each ΔP condition. Each measurement was repeated five times, and the average value was used for analysis. Importantly, data are recorded only after the gas‐side surface of the graphite is fully wetted to ensure full utilization of available flow paths. As depicted in Figure 3c, titanium presents a higher water flux. The water flux of both materials increases monotonically with the pressure differential and exhibits a good linear dependence.

#### 2.1.5 Bubble Pressure Measurement

Because the WTP must prevent O_2_ leakage into the coolant, its bubble pressure must be quantified. During the test, water is continuously supplied to the water chamber (Figure 3b) to emulate coolant flow, and the outlet pressure is recorded as the water‐side pressure. Oxygen is then introduced to the gas chamber, and its pressure is increased gradually until bubbles first appear in the water chamber. The difference between the gas‐side pressure and the water‐side pressure at breakthrough is taken as the bubble pressure. Figure 3d shows that the graphite sample demonstrates superior sealing performance with a higher bubble pressure. Each bubble pressure measurement was repeated five times, and the average value was reported.

### 2.2 Cathode Plate and Stacks Design

**FIGURE 4:**
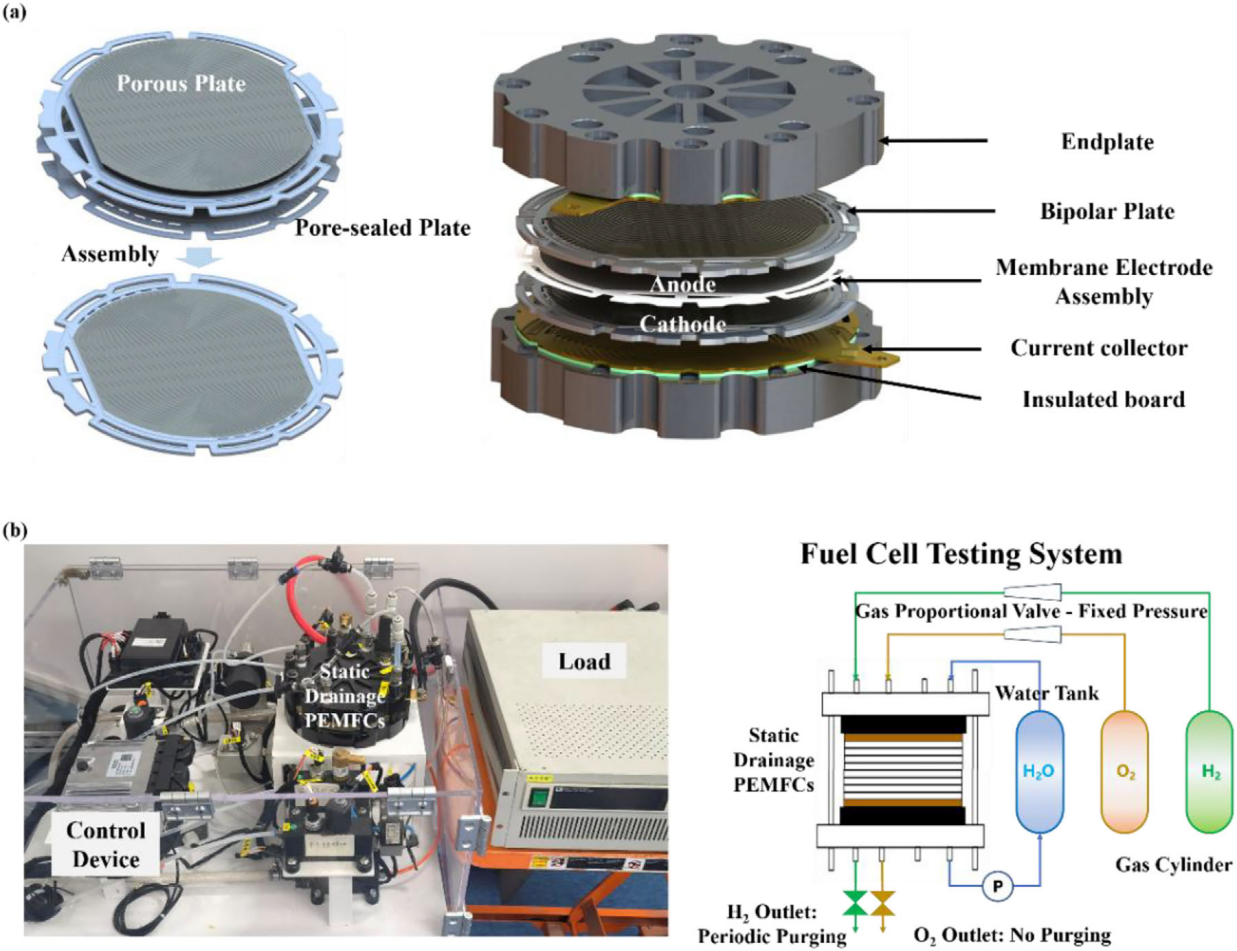
(a) Bipolar plate structure and PEMFC stacks’ structure; (b) Fuel cell testing system.

As shown in Figure 4a, the cathode plate is fabricated from a combination of a porous plate and a pore‐sealed plate. The outer frame is made of the pore‐sealed plate and incorporates the sealing features as well as the ports for the gas and coolant chambers. To prevent irreversible flooding under abnormal operating conditions, a cathode outlet was retained for emergency purging. The internal reaction zone was composed of the porous plate region. The porous plate used in the experiments has a thickness of 1.5 mm, while the pore‐sealed plate also has a thickness of 1.5 mm. The porous plate is joined to the sealing plate via a welding process. Prior to assembly, a 2 mm‐wide edge region of the porous plate was compressed by a hot‐pressing process to reduce pore size and achieve edge sealing of the porous structure. The contact resistance between WTP and GDL was 5.18 mΩ·cm^2^. The assembly torque was 5 N·m, corresponding to an assembly pressure of 1 MPa. In addition, the porous titanium substrate was subjected to a 50 nm gold coating treatment. Flow channels are machined on the porous plate cathode face with a rib‐to‐groove ratio of 1:1, a channel width of 2 mm, and a total active area of 168 cm^2^. Leak testing verified the sealing integrity of the bonded joint.

The static drainage PEMFC stack is assembled with two single cells. As illustrated in Figure 4a, the stack was oriented with the flow‐field direction perpendicular to gravity and the cathode placed beneath the MEA. The current flow field adopts a parallel configuration with rib–channel structures, where gravity assists in guiding water droplets generated on the GDL surface toward the channel regions and subsequently onto the surface of the WTP. The pore size of the WTP is in the micrometer range, where water transport is primarily governed by capillary forces, while gravitational effects are negligible. Once the droplets contact the porous plate, they are drawn into the plate by capillary forces and then discharged.

### 2.3 Cell Performance Testing

The test system illustrated in Figure 4b was established. To verify whether the WTPs can continuously and stably discharge the water generated on the cathode side, the cathode side operated in a dead‐end configuration. A purge was initiated only when the stack voltage decreased by more than 20%. The anode was also dead‐ended and purged for 0.5 s every 180 s. Stack performance tests were conducted for more than 30 h.

Given the stringent efficiency requirements for underwater and space power systems, the test current density was set to 0.5 A·cm^−2^ to favor higher energy‐conversion efficiency. To verify the water drainage capacity of the WTP under high current density operating conditions, a current density of 1 A·cm^−2^ was employed as an additional test condition to further assess the operational stability of the fuel cell stack. Both oxygen and hydrogen were supplied with no external humidification. Inlet pressures of H_2_ and O_2_ were maintained at 100 kPa gauge pressure under fixed‐pressure control for the anode and cathode sides, respectively. Separate proportional valves were installed at the anode and cathode inlets to regulate the gas pressures. The test temperature was 65°C. Coolant flow rate was 200 mL·min^−1^, and the water chamber pressure was regulated to 80 kPa via a back‐pressure valve.

## 3 Modeling and Model Verification

Considerable research has been conducted on the capillary behavior and water transport characteristics of porous structures, particularly in fuel cell applications, where investigations have primarily focused on GDL. Simulation methods include the Volume of Fluid (VOF) method, Smoothed Particle Hydrodynamics (SPH), Lattice Boltzmann Method (LBM), and Pore Network Model (PNM) [42, 43, 44]. WTPs are typically sintered from powders, resulting in lower porosity, smaller pore sizes, and greater variability in pore connectivity. Therefore, appropriate modeling approaches are required to investigate the effects of pore‐scale structural parameters on capillary transport behavior within WTPs. As a representative pore‐scale modeling approach, PNM describes porous media using interconnected pore–throat networks while preserving essential pore‐scale characteristics. This feature enables the quantitative analysis of the effects of structural parameters, making PNM a more suitable approach for investigating water transport in WTPs.

To predict the drainage and sealing performance of WTPs, PNM was employed to simulate the porous structures. The capillary flow behavior within complex micro‐nano apertures was analyzed to reveal the influence of pore‐scale parameters on WTP performance. The model was modified by considering key structural parameters including pore shape, tortuosity, and coordination‐number distribution. Further validation was conducted using statistical data derived from CT images, as well as experimental measurements of water flux and bubble pressure of WTP samples.

### 3.1 Numerical Model for Porous Plates

#### 3.1.1 Governing Equations

A porous medium can be idealized as a three‐dimensional network of capillaries formed by interconnecting pores. For an incompressible Newtonian fluid flowing in a long, cylindrical capillary, the dynamics are described by Newtonian (Bosanquet‐type) capillary flow [45]:

(1) $$\rho \;\left[zz^{\prime \prime }+{\left(z^{\prime }\right)}^{2}\right]=\frac{2}{R}\;\sigma_{L}\cos\theta_{c}-\frac{8}{R^{2}}\mu zz^{\prime }-\rho gz$$

where, *ρ* is the fluid density, *z* is the penetration length, *σ_L_* is the surface tension, *θ_C_* is the three‐phase contact angle governed by Young's law, *µ* is the dynamic viscosity, *R* is the effective capillary radius, and *g* is the gravitational acceleration.

The left side of the equation represents the inertial force, while the three terms on the right side correspond to the capillary force, viscous resistance, and gravity, respectively.

Dreyer et al. [46] partition the capillary filling process into three regimes: (i) inertia‐dominated, (ii) inertia–viscous, (iii) viscous‐dominated. For the water permeation rate considered in this work, the regime of interest is the late‐time stage after the porous matrix becomes fully saturated with water. Over this time scale, inertial effects are negligible and the flow is governed by a balance of capillary and viscous forces. The Lucas–Washburn (L–W) model is used to describe this regime [47, 48]:

(2) $$\frac{dz}{dt}=\frac{\Delta P\left(R^{2}+4\varepsilon R\right)}{8\mu z}\;$$

where *ΔP* is the total pressure difference between the two capillary ends, *ε* is the slip coefficient, *µ* is the viscosity.

Under a prescribed pressure drop, the relationship between volumetric flow rate *Q* and *ΔP* follows the Hagen–Poiseuille law [49]:

(3) $$Q=\frac{\pi R^{4}\Delta P}{8\mu z}\;$$

For the bubble pressure, the pore sizes are in the micro/nanoscale, so gravity is neglected. The capillary pressure equals the pressure difference between the gas and water chambers. The Young–Laplace equation is used to obtain this pressure difference (i.e., the bubble pressure *P_b_*) [50]:

(4) $$P_{b}=\Delta P=\frac{2\sigma_{L}\cos\theta_{c}}{R}\;$$

#### 3.1.2 Pore‐Network Architecture

**FIGURE 5:**
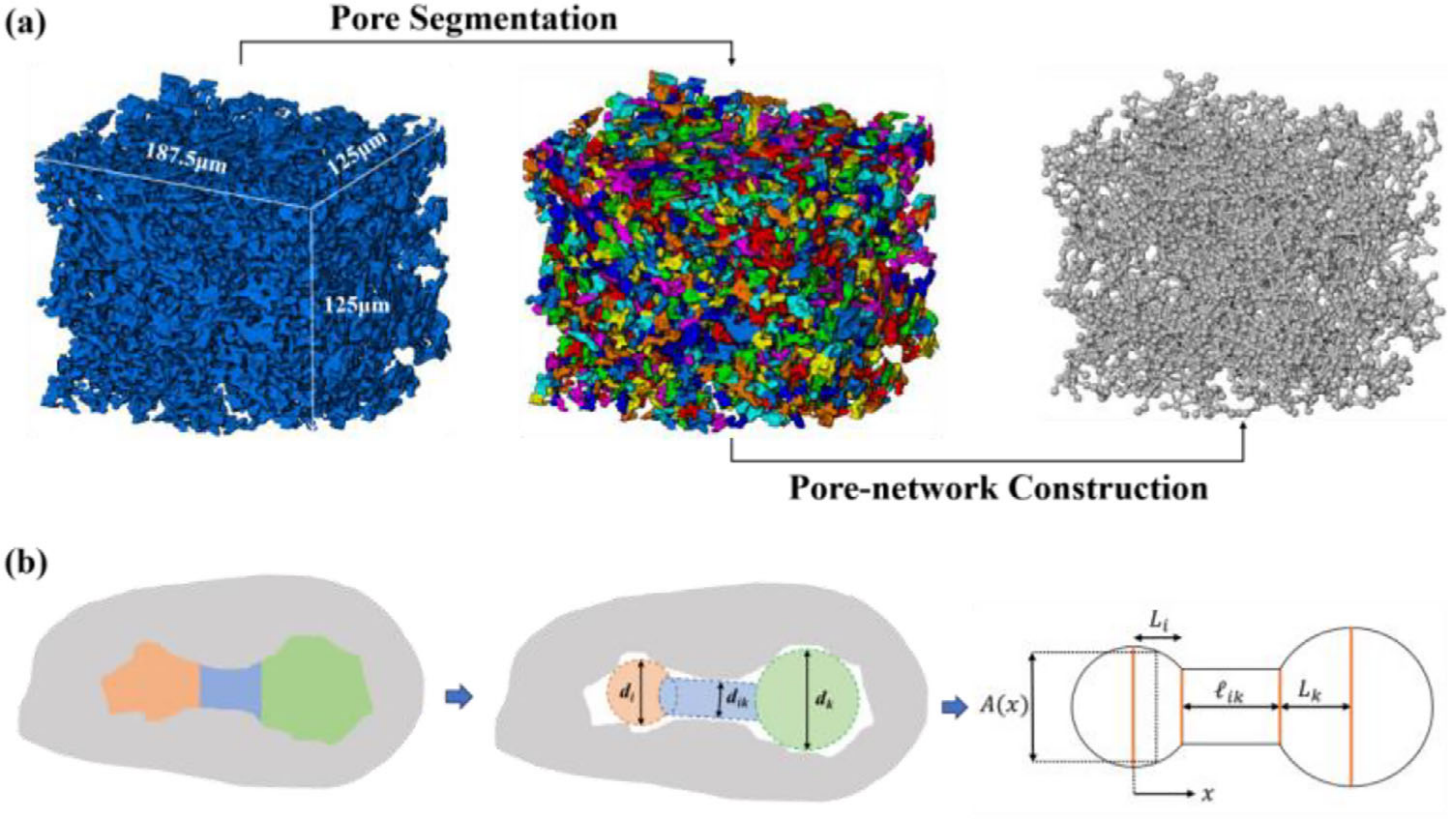
(a) Establishment of PNM by X‐CT image post‐processing; (b) Equivalent process of pore shape.

In the pore‐network model, a three‐dimensional network is constructed by connecting nodes with negligible volume through pore throats. However, pore‐scale parameters, including pore connectivity, spatial distribution, and pore size, need to be independently assigned. In general, X‐CT images can be segmented and simplified to construct a pore network that approximates the material's true internal structure. As shown in Figure 5a, CT slices were post‐processed in *Avizo* to identify and extract the pore phase. Subsequently, a subvolume of 600 × 400 × 400 voxels was selected, and pore segmentation was performed to determine the spatial locations of pore nodes. The connectivity between neighboring pores was then identified, and throats were generated to connect adjacent pores. Consequently, a ball‐and‐stick pore‐network skeleton was obtained, while pore diameter, throat diameter, and pore geometry remained to be determined.

Using the Sub‐Network of an Over‐segmented Watershed (SNOW) method [51], the segmented pore space is parameterized. As shown in Figure 5b, pores and throats in the pore‐network model are simplified as spheres and cylinders, respectively. Evidently, this equivalence is inaccurate for irregularly shaped pores [52]. To incorporate the influence of noncircular geometry, the formula for hydraulic resistance must be modified. Assuming a gradually varying cross‐sectional shape, the pressure *P_c_* drop along a pore channel under steady‐state conditions can be computed by the following expression:

(5) $$-\frac{1}{Q}\;\frac{dP_{c}}{dx}=\mu \left[16\pi^{2}\cdot \frac{I_{p}}{A{\left(x\right)}^{2}}-\frac{2\rho Q}{\mu }\cdot \frac{dA\left(x\right)/dx}{A{\left(x\right)}^{3}}\right]$$

where, *Q* is the volumetric flow rate, *I_p_* is the specific polar moment of inertia, *A(x)* is the cross‐sectional area of the conduit. After integrating and simplifying the above equation, we can further calculate the pressure difference(*∆P_ik_*) between pores *k* and *i*:

(6) $$\frac{\Delta P_{ik}}{Q}=16\pi^{2}\mu \int \frac{I_{p}}{A{\left(x\right)}^{2}}dx+\rho Q\cdot \left(\frac{1}{A_{k}^{2}}-\frac{1}{A_{i}^{2}}\right)$$

The first term on the right‐hand side represents the frictional loss, while the second term corresponds to the inertial loss. Since the inertial loss is typically much smaller than the frictional loss, it can be neglected, yielding:

(7) $$\frac{\Delta P_{ik}}{Q}=16\pi^{2}\mu \int \frac{I_{p}}{A{\left(x\right)}^{2}}dx$$

Denoting the hydraulic conductance by *g_ik_*, we obtain the volumetric flow rate *Q* [52]:

(8) $$Q=g_{ik}\;\cdot \Delta P_{ik}$$

Further decomposing into *pore_i_*, *poer_k_*, and *throat_ik_* hydraulic conductance:

(9) $$\begin{array}{ccc} \frac{1}{g_{ik}} & = & 16\pi^{2}\mu \left[{\left(\int \frac{I_{p}}{A_{i}{\left(x\right)}^{2}}dx\right)}_{pore\;i}+{\left(\int \frac{I_{p}}{A_{ik}{\left(x\right)}^{2}}dx\right)}_{throat\;ik}\right.\\ & & \left.+\;{\left(\int \frac{I_{p}}{A_{k}{\left(x\right)}^{2}}dx\right)}_{pore\;k}\right] \end{array}$$

In this model, variations in the moment of inertia *I_p_* and cross‐sectional area *A(x)* ^2^ of pores and throats along their length are considered. It enables approximate calculations for irregular shapes, better aligns with actual pore geometries, and is more accurate than using pure cylindrical to represent capillaries shape.

For the throat axial geometry, straight throats are assumed by default, which ignores actual tortuosity and introduces additional error. Using *Avizo*, the tortuosity distribution of the throats was quantified; this distribution is incorporated into the conductance calculation by replacing the straight throat length with the measured tortuous length.

#### 3.1.3 Boundary Conditions

The numerical domain was configured using the same parameters as the experimental samples. The working fluid was water at ambient temperature (25°C), with a viscosity of 0.890×10^−3^ Pa·s. For water flux calculations, all hydrophilic pathways (contact angle < 90°) were considered as effective flow pathways. By default, water transport was restricted to the x‐direction, with no water discharge allowed through the other boundary surfaces. For bubble pressure determination, a gas‐invasion process was simulated: an initial gas pressure of 10 kPa was applied at the min‐x surface and then increased gradually until breakthrough occurs at the max‐x surface; the gas pressure at breakthrough was taken as the bubble pressure.

The contact angle within the porous domain was assumed to be spatially heterogeneous. In situ X‐CT observations by Ibekwe et al. [53] demonstrated that the contact‐angle distribution in porous media approximately follows a normal distribution, which has been further supported by subsequent studies. Therefore, in this model, contact angles were assigned to pores according to a truncated normal distribution for bubble pressure simulations.

#### 3.1.4 Equivalence of pore geometry

**FIGURE 6:**
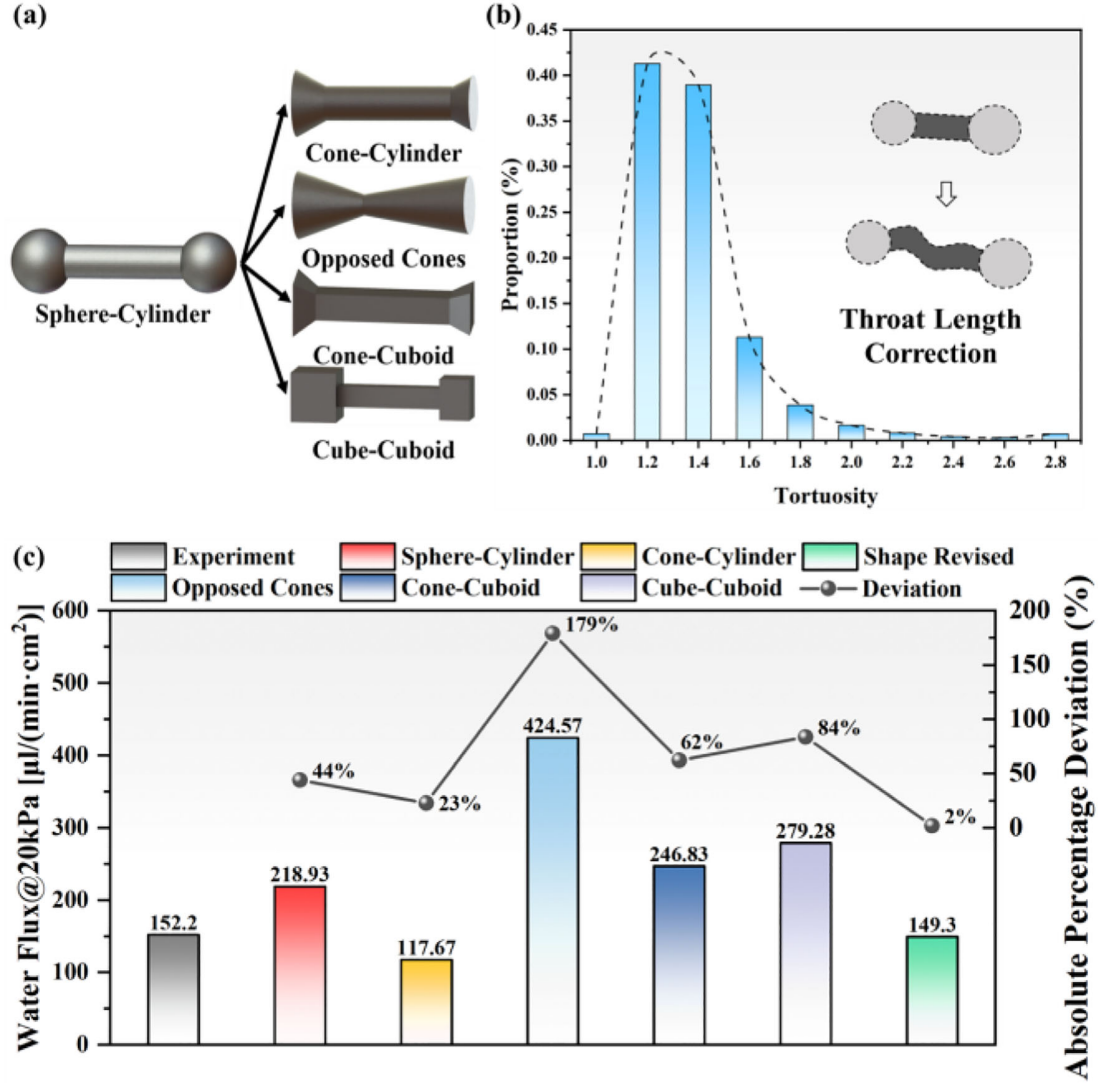
(a) Different pore‐throat shape combinations; (b) Tortuosity distribution; (c) Simulation results of water flux for models with different pore‐throat shapes.

The influence of pore shape on water fluxes was investigated. As shown in Figure 6a, five pore‐throat shape combinations were evaluated: (i) sphere‐cylinder, (ii) cone‐cylinder, (iii) opposed cones, (iv) cone‐cuboid, and (v) cube‐cuboid. By means of Equation 9, the hydraulic conductance corresponding to five distinct geometries can be separately derived via integration. This parameter serves as the shape correction factor for the hydraulic conductance of actual pores. The resulting water fluxes obtained with these shape‐correction schemes are summarized in Figure 6c. Shape correction had a pronounced influence on the predicted water flux, with a maximum deviation of 178.9%. Among the tested geometries, the cone–cylinder structure, with a deviation of 23%, showed the best agreement with the pore morphologies observed in porous samples. For cone‐cylinder, the associated hydraulic conductance remains overestimated. Based on the observation of Akbari et al. [54]. that the pore–throat transition region contributes significantly to hydraulic resistance, an additional transition‐region resistance correction factor was introduced for the cone–cylinder geometry. Furthermore, considering the tortuous nature of inter‐pore connections, the throat length was corrected accordingly, as shown in Figure 6b. The tortuosity distribution of the throats was extracted from *Avizo* and converted into a probability distribution, which was subsequently incorporated into the model as a correction parameter for throat length. The final results (Figure 6c) indicate that jointly correcting the shape factor and throat length, two key pore‐scale parameters, substantially improves the model's predictive accuracy.

#### 3.1.5 Construction of Pore‐Network Structure

To obtain the optional range of pore parameters, pore networks with different structural characteristics need to be constructed. However, in CT‐based pore‐networks, the pore structure is fixed. Modifying the diameter independently will lead to excessive engulfment and overlapping of adjacent pores, preventing the formation of throats at these locations and affecting the calculation. Therefore, it is necessary to construct a structure similar to the CT model, enabling overlapping pores to be repositioned rationally when adjusting average pore diameter individually.

**FIGURE 7:**
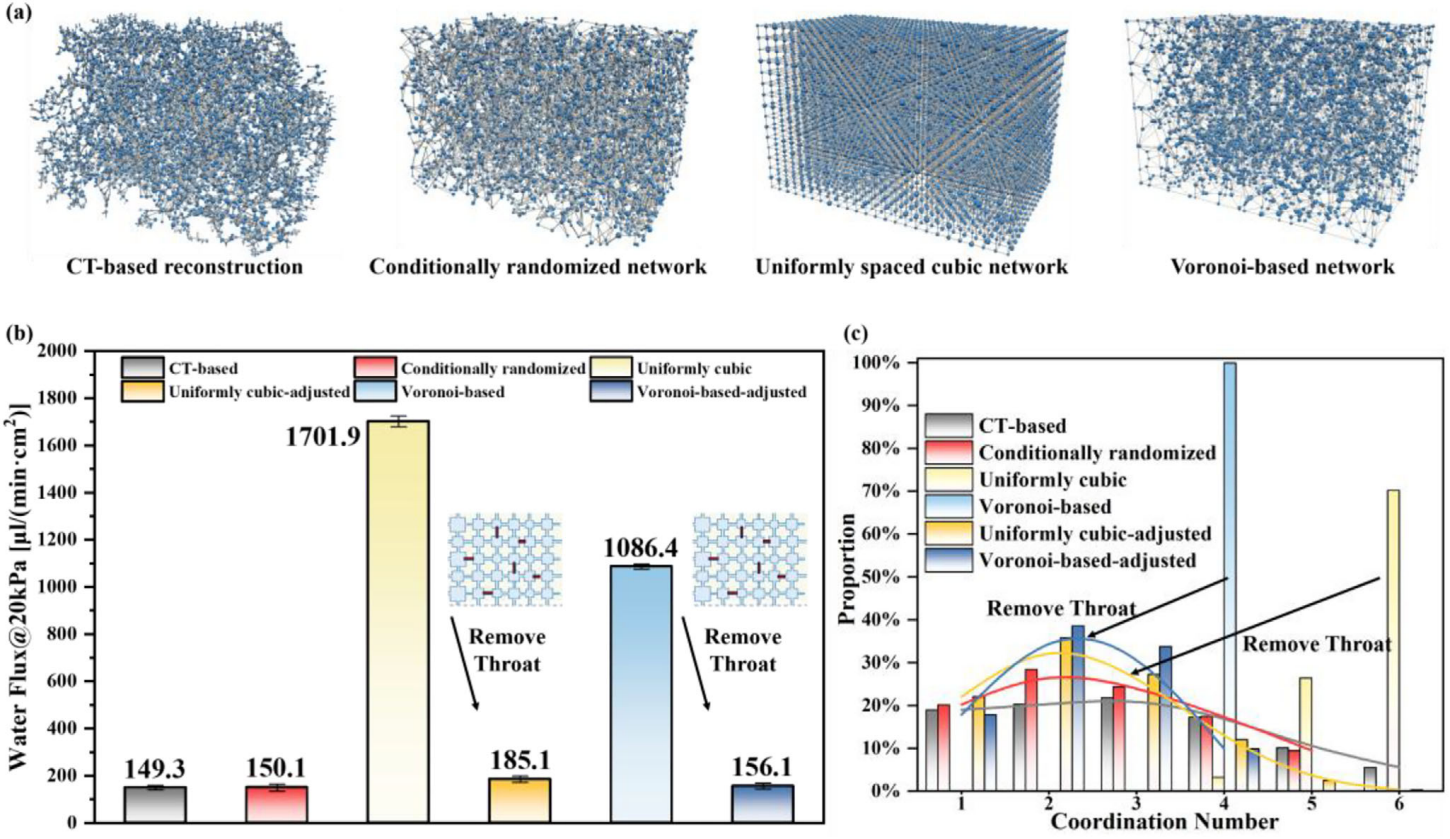
(a) Different network‐construction strategies; (b) Simulation results of water flux for models with different network structure; (c) The coordination‐number distribution of different network structure.

As shown in Figure 7a, four network‐construction strategies were examined for building the PNM: CT‐based reconstruction, a conditionally randomized network, a uniformly spaced cubic network, and a Voronoi‐based network. The CT‐based reconstruction was used as the reference, and pore‐scale parameters extracted from it, including porosity, pore‐size distribution, throat diameter distribution, tortuosity distribution, and contact‐angle distribution, served as benchmark inputs for the other network constructions. In the cubic network, pore centers were arranged on a regular lattice with equal spacing, and each interior pore was connected to six neighbor pores. The Voronoi‐based network was generated through a three‐dimensional uniform Poisson point process. Unlike the cubic network, the pore centers in the Voronoi network are non‐uniformly distributed and the inter‐pore distances vary, with each pore connected to four neighboring pores. As shown in Figure 7b, the three network topologies yield markedly different water flux predictions despite sharing identical pore‐size distributions and porosity values, underscoring the substantial impact of network structure on water transport calculations. Each data point represents the average value obtained from ten independent simulations.

The coordination‐number distribution is commonly used to characterize pore‐network topology. As seen in Figure 7c, the coordination numbers of the CT‐based reconstructed network are mainly distributed between 1 and 4, accounting for more than 80% of the pores. By contrast, the coordination numbers of the cubic and Voronoi networks are mainly concentrated around 6 and 4, respectively, resulting in significantly higher water fluxes. To isolate the influence of coordination number, a randomly positioned pore‐center network was generated while constraining its coordination‐number distribution to match that of the CT‐based reconstruction. In addition, throat pruning was performed on the cubic and Voronoi networks to reduce their coordination numbers without modifying other pore‐scale parameters.

Taking the probability distribution of coordination numbers obtained from CT‐based reconstruction networks as the benchmark, the overlap coefficient (OC) was utilized to quantify the similarity between distributions. The OC ranges from 0 to 1, with higher values indicating greater agreement between distributions. In addition, the chi‐square goodness‐of‐fit test was applied to evaluate the statistical significance of distribution discrepancies at a significance level α = 0.05. When the calculated chi‐square statistic exceeds the corresponding critical value determined by the degrees of freedom, a statistically significant difference exists between the two coordination‐number distributions. The test results are summarized in Table 1.

**TABLE 1:** OC and Chi‐Square Test Results.

	OC	Chi‐square Statistic	Critical Value	Statistical Significance (α = 0.05)
Conditionally randomized	0.8787	0.037	9.488	No Significant Difference
Uniformly cubic‐adjusted	0.7594	0.2609	11.07	No Significant Difference
Voronoi‐based‐adjusted	0.6983	0.2618	7.815	No Significant Difference
Uniformly cubic	0.1911	8.4752	7.815	Significant Difference
Voronoi‐based	0.1736	3.902	3.841	Significant Difference

The random structure achieves the highest OC value of 0.8787 among all comparison samples with an extremely low chi‐square value, demonstrating excellent consistency between its coordination‐number distribution and that of the CT‐based reconstruction. After structural modification, the cubic and Voronoi networks exhibit moderate distribution overlap with OCs of 0.7594 and 0.6983, respectively. No significant differences are detected via the chi‐square test. Although their overall coordination‐number distributions follow similar trends to the CT‐based network, slight deviations remain in the proportion of pores with different coordination numbers. In contrast, without structural modification, both the cubic and Voronoi networks exhibit OC values below 0.2, indicating negligible overlap with the CT‐derived coordination‐number distribution. The chi‐square test confirms statistically significant distribution differences relative to the CT‐based reconstruction, revealing drastically distinct pore connectivity topological characteristics for the two networks.

As shown in Figure 7b,c, when the coordination‐number distribution of the randomized network resembles that of the CT‐based case, the predicted water flux likewise approaches the CT‐based result. After structural adjustment, the cubic and Voronoi networks also produce higher water fluxes comparable to the CT‐based network. These findings indicate that the coordination of the pore network is a non‐negligible factor in water‐flux prediction. Eventually, a conditionally randomized network was chosen, whose coordination‐number distribution is more consistent with that of the CT‐based reconstruction.

### 3.2 Model Verification

Water flux and bubble pressure tests as mentioned in Section 2.1 were performed on the porous samples. The experimentally measured water fluxes of the porous sample were 152.2 µL·cm^−2^ ·min^−1^ for graphite and 2176.4 µL·cm^−2^ ·min^−1^ for titanium. The bubble pressures were 96 kPa for graphite and 50 kPa for titanium. These experimental results were used as the evaluation criteria for the validation of the pore‐network model.

**FIGURE 8:**
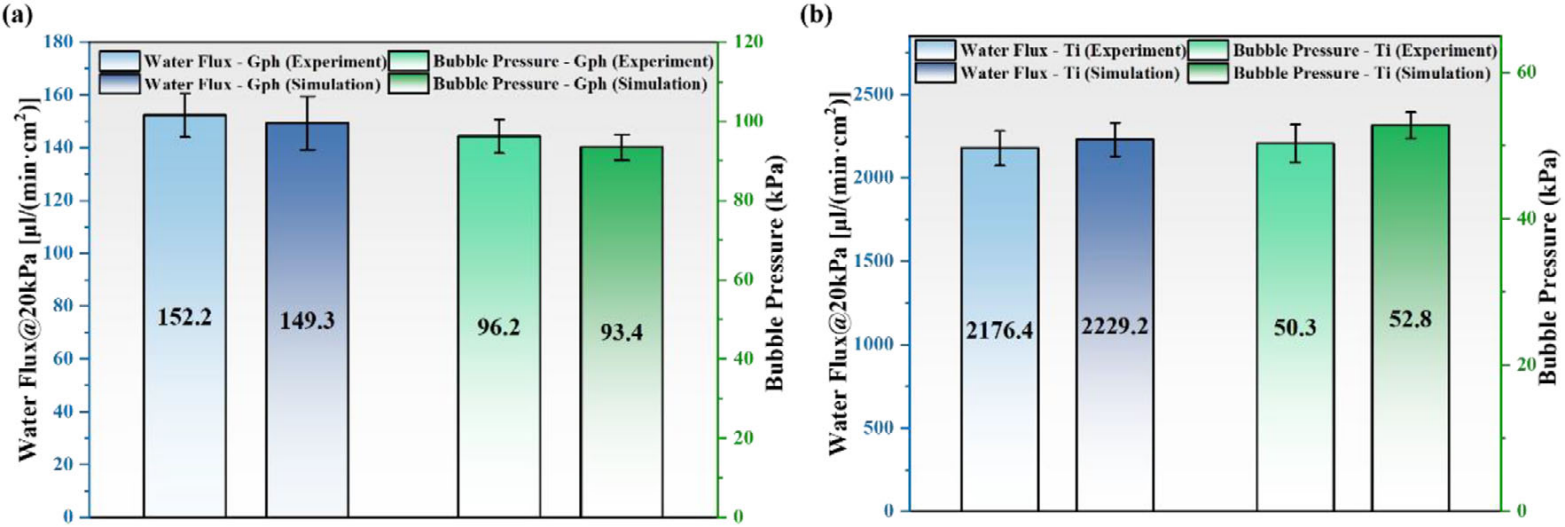
Comparison of simulated and experimental values of (a) Graphite; (b) Ti.

Because the pore‐scale parameters in the model (e.g., pore diameter and contact angle) were sampled from prescribed probability distributions, the predictions exhibit stochastic variability. Twenty independent realizations were performed, and the statistical characteristics of the predicted results were analyzed. As shown in Figure 8a, the ensemble‐mean water flux was 149.3 µL·cm^−2^·min^−1^ and the mean bubble pressure was 93.4 kPa. The deviations between the model predictions and experimental measurements were 4.89% for water flux and 6.11% for bubble pressure. Based on the pore parameters of titanium, a predictive model was established following the same procedure. The deviations between the simulation results and the experimental data were 2.4% for water flux and 5.6% for bubble pressure (Figure 8b). The results indicate that the model exhibits high consistency with the experimental data and can accurately predict the water flux and bubble pressure of porous materials.

## 4 Results and Discussion

### 4.1 Effect of Temperature

**FIGURE 9:**
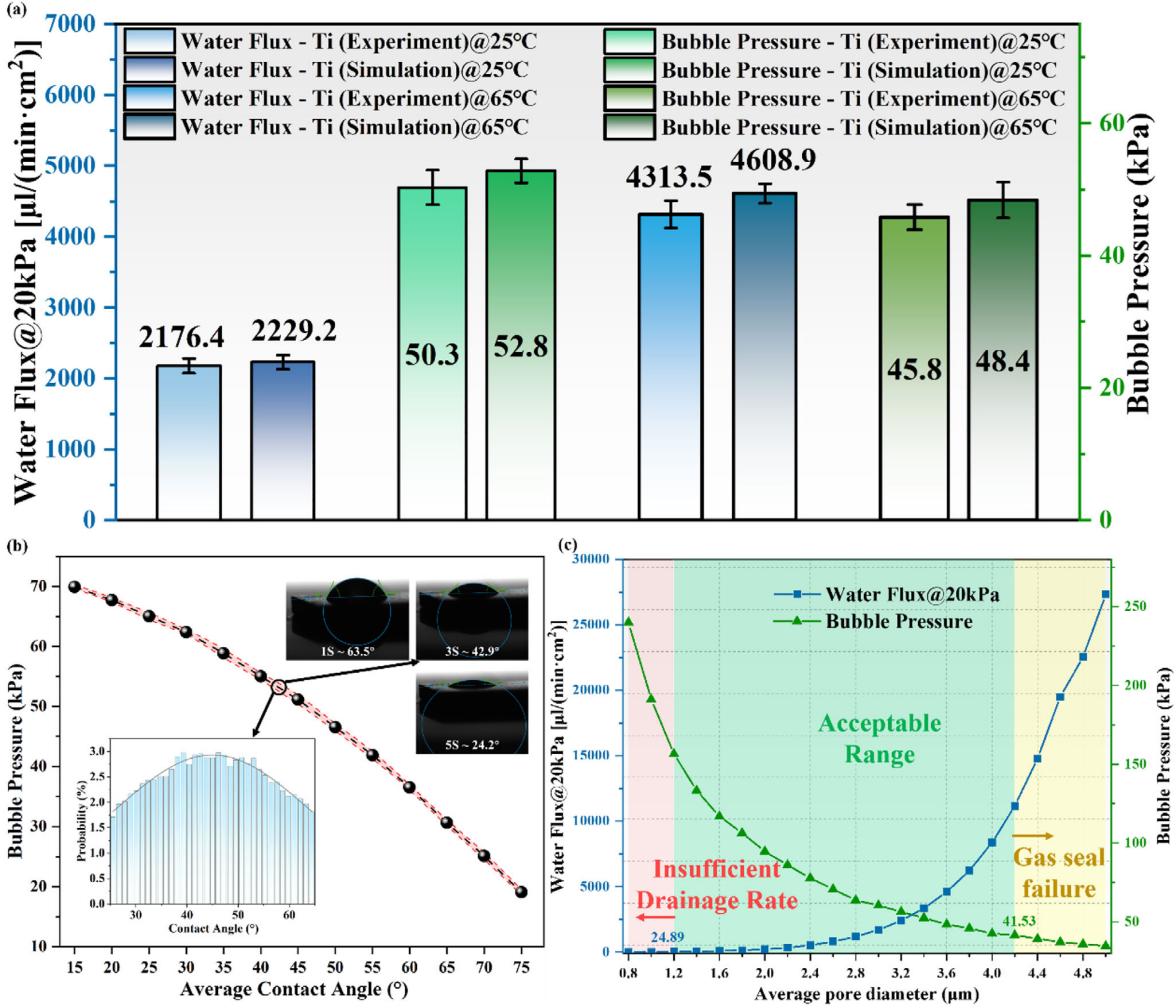
(a) Comparison of simulated and experimental data for porous titanium under different temperatures; (b) The variation of bubble pressure with the average contact angle; (c) The variation of water flux, bubble pressure with the average pore diameter.

As shown in Figure 9a, a porous titanium WTP sample was investigated at both 25°C and 65°C, with experimental measurements of water flux and bubble pressure, accompanied by numerical simulations for validation. The results indicate that increasing the temperature from 25°C to 65°C significantly enhanced water flux. The experimentally measured water flux increased from 2176.4 to 4313.5 µL·cm^−2^·min^−1^, while the simulation result increased from approximately 2229.2 to 4608.9 µL·cm^−2^·min^−1^. Meanwhile, the bubble pressure slightly decreased with increasing temperature, from approximately 50.3 to 45.8 kPa in the experiment, and from 52.8 to 48.4 kPa in the simulation. At 65°C, the relative errors between simulation and experiment are 6.85% for water flux and 5.68% for bubble pressure, respectively. Overall, the simulation results show good agreement with the experimental data under both temperature conditions. No significant reduction in predictive accuracy is observed at the elevated temperature of 65°C, indicating that the proposed model can reliably capture the temperature‐dependent two‐phase transport behavior within the porous structure.

The increase in temperature from 25°C to 65°C induces significant changes in the relevant fluid properties. The dynamic viscosity of water decreases from approximately 0.89 to 0.47 mPa·s, while the surface tension decreases from 72 to 66 mN·m^−1^. These variations directly affect viscous resistance and capillary forces within the porous medium. The reduction in viscosity decreases the flow resistance within the porous network, thereby enhancing water transport. Meanwhile, the reduction in surface tension weakens capillary pressure. According to the Young–Laplace relationship, reduced interfacial tension lowers the capillary pressure barrier, facilitating gas penetration into liquid‐filled pores and consequently resulting in a slight decrease in bubble pressure.

### 4.2 Influence of Pore Surface Hydrophilicity

In this section, the influence of hydrophilicity on the sealing performance of porous titanium was investigated. In the present model, a truncated normal distribution as shown in Figure 9b was adopted when assigning contact angles to individual pores. The range of surface contact angle distribution for the sample was determined via a contact angle measurement system. The contact angles measured immediately following droplet deposition onto the material surface and just prior to complete liquid absorption were adopted as the upper and lower limits, respectively. The contact angle measured on a flat and smooth titanium plate was adopted as the mean value of the distribution. The influence of hydrophilicity on bubble pressure was evaluated by varying only the mean contact angle while maintaining all other pore‐scale parameters unchanged.

As shown in Figure 9b, the bubble pressure increases gradually with decreasing contact angle. The results demonstrate that enhancing pore hydrophilicity can effectively improve the bubble pressure without altering other parameters. For hydrophilic pores, the bubble pressure is also affected by the average pore diameter.

### 4.3 Optimal Selection of Pore Diameter

In this section, titanium was used as the prediction object. We obtained the variation curves of water flux and bubble pressure with average pore diameter. The physical parameters used in the model were obtained at 65°C to better represent the actual operating conditions inside the fuel cell stack. The average pore diameter is treated as the sole control variable. With the network topology fixed and the number of pores unchanged, only the average pore diameter is shifted to represent porous media of different compaction levels.

Figure 9c illustrates the variations in water flux and bubble pressure with average pore diameter. As the average diameter increases, water flux rises rapidly whereas bubble pressure decreases, revealing a clear trade‐off. Because WTPs must simultaneously deliver high drainage rates and a sufficiently large bubble pressure, an average pore diameter selection range is required. The pressure differential between the cathode and coolant is nominally 20 kPa. Considering pressure‐control delays and unavoidable operational fluctuations, the bubble pressure should exceed the applied pressure difference by an adequate margin. By introducing a safety factor of 2, a minimum bubble pressure requirement of ≥40 kPa was established to prevent gas leakage during operation. At the rated current density of 0.5 A·cm^−2^, the water production rate is 2.79 µL·cm^−2^·min^−1^. Considering that only approximately half of the nominal interfacial area contributes effectively to water transport, and that the effective drainage area may further decrease after reaching a quasi‐steady drainage state, a conservative safety factor of 10 was applied. Accordingly, the required water flux was determined to be ≥27.9 µL·cm^−2^·min^−1^. By simultaneously satisfying the water flux and bubble pressure criteria, an acceptable average pore diameter range of 1.2–4.2 µm was identified for WTP design.

### 4.4 Performance of the PEMFC Stacks

#### 4.4.1 Water Drainage Capacity of WTPs

For the tested porous plate samples, increasing the average pore diameter can enhance water drainage capacity while ensuring the bubble pressure exceeds 40 kPa. Thus, this study selected porous titanium, characterized by superior mechanical properties and corrosion resistance, to fabricate the optimized WTP. Titanium powder with a particle size of 38 µm was selected for compaction and sintering to form porous titanium plates. Testing on the porous titanium sample revealed an average pore diameter of 3.6 µm. Experimental results showed a water flow rate of 2229.2 µL·cm^−2^·min^−1^ at a pressure difference of 20 kPa, with a bubble pressure of 50.3 kPa. To evaluate the drainage and sealing performance of the porous plate using in the fuel cell, 2‐cell stacks were assembled using the cathode plate described in Section 2.2.

Titanium powders with particle sizes of 5–10 µm and approximately 50 µm were sintered to fabricate two porous plates, denoted as WTP01 and WTP02, with average pore diameters of approximately 0.8 and 5.1 µm, respectively. WTP01 exhibited a lower water flux and higher bubble pressure, whereas WTP02 showed a higher water flux and lower bubble pressure. As summarized in Table 2, the water flux and bubble pressure of the model‐guided optimized WTP, WTP01, and WTP02 were experimentally characterized and compared. Three fuel cells assembled with WTP01, WTP02 and SP replacing the optimized WTP were used as control groups under identical configurations.

**TABLE 2:** Water flux and bubble pressure of WTP, WTP01, and WTP02.

	Average pore diameter (µm)	Water flux@20kPa‐65°C (µL·cm^−2^·min^−1^)	Bubble pressure‐65°C (kPa)
WTP	3.6	4313.5	45.8
WTP01	0.8	3.67	225.3
WTP02	5.1	24312.2	14.6

Under dead‐ended cathode operation, as illustrated in Figure 4b, long‐duration performance tests were conducted to evaluate the drainage capability and sealing reliability of different WTP designs. A current density of 0.5 A·cm^−2^ was first selected to compare the optimized WTP with the SP, aiming to demonstrate the improvement in cathode water management enabled by the WTP. Subsequently, the operating current density was increased to 1 A·cm^−2^ to further evaluate the drainage capability of the optimized WTP under high water‐generation conditions and compare its performance with WTP01 and WTP02.

**FIGURE 10:**
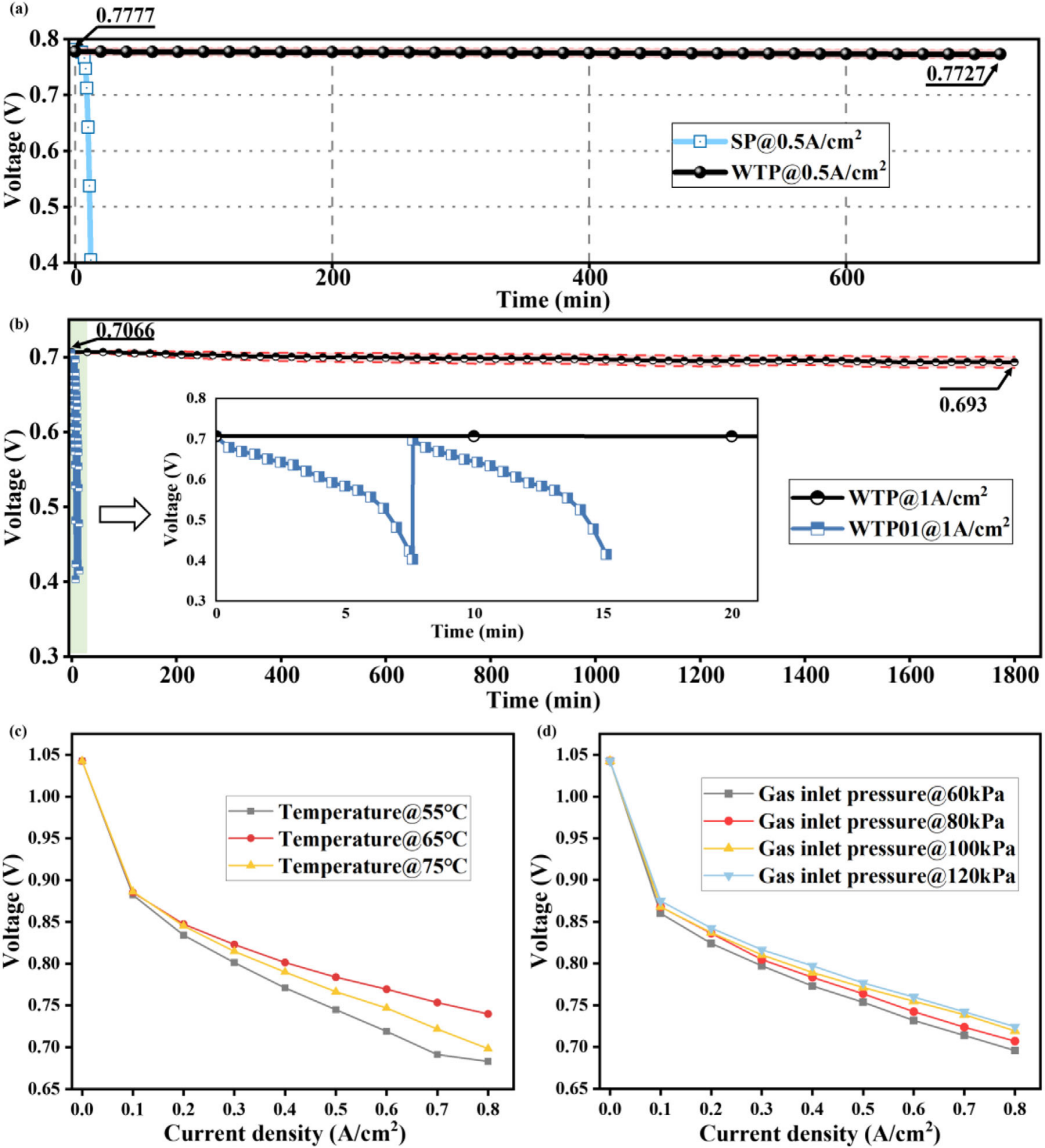
(a) Performance of the stacks (with SP and WTP) during 12‐h operation; (b) Performance of the stacks (with WTP and WTP01) during 30‐h operation; (c) Effect of different temperature and (d) gas inlet pressure on the stacks’ performance.

With no cathode purging, the gas chamber would otherwise fill with water within about 10 min in the absence of WTPs. Figure 10a plots the stacks voltage vs. time. After 12 h, the mean voltage decreased by 5 mV, corresponding to a 0.64% reduction in performance, demonstrating that the deployed WTP effectively removed product water within the cell. The fuel cell assembled with SP experiences rapid flooding, and the cell voltage quickly drops below 0.4 V within 12 min.

Since the bubble pressure of WTP02 was 14.6 kPa, oxygen leakage into the coolant occurred under the applied pressure difference of 20 kPa between the cathode and coolant sides. Therefore, further electrochemical performance tests were not conducted. As shown in Figure 10b, after 30 h of operation, the average cell voltage decreased by 13.6 mV, corresponding to a performance degradation of 1.92%. For the fuel cell assembled with WTP01, the cell voltage decreased rapidly from 0.699 to 0.401 V after approximately 7 min of operation, indicating severe cathode flooding caused by insufficient water removal capability. After applying a 0.1 s purge to remove accumulated water from the cathode, the cell voltage recovered to 0.697 V, further confirming that the performance degradation was mainly associated with water accumulation.

These results demonstrate that WTPs with insufficient drainage capability or insufficient gas retention capability cannot simultaneously satisfy the requirements of dead‐ended cathode operation. The model‐guided optimization strategy helps identify an appropriate balance between water flux and bubble pressure, which may contribute to improving the stability of dead‐ended operation.

Furthermore, compared with hydrogen–oxygen fuel cell systems requiring oxygen recirculation or frequent pulsed purging, the proposed configuration may potentially reduce auxiliary power demand and reactant loss by avoiding continuous gas circulation and minimizing purge operations.

#### 4.4.2 Sensitivity of Operating Conditions for the Stacks

The influence of operating conditions on stacks performance is also significant. Therefore, to achieve better stacks performance, tests were conducted under different operating conditions. This study mainly analyzes the effects of different temperature and inlet gas pressure. The parameter sensitivity test settings are shown in Table 3.

**TABLE 3:** Configuration of sensitivity experiments for stacks operating conditions.

	Temperature (°C)	H_2_ inlet pressure (kPa)	O_2_ inlet pressure (kPa)	Coolant pressure (kPa)
1	55	100	100	80
2	65	100	100	80
3	75	100	100	80
4	65	60	60	40
5	65	80	80	60
6	65	120	120	100

As shown in the Figure 10c, the stacks performance first increases and then decreases with increasing temperature, with the best performance achieved at 65°C. When the temperature is below 65°C, increasing temperature significantly enhances the oxygen reduction reaction (ORR) kinetics and reduces ohmic losses associated with proton conduction. In addition, the saturated vapor pressure of water is relatively low, so the dilution effect of water vapor on the oxygen partial pressure at the cathode is limited. As a result, the overall cell performance increases with temperature. However, when the temperature exceeds 65°C, the saturated vapor pressure of water increases markedly with temperature. Under a closed system without gas purging, water vapor continuously accumulates and reaches saturation, leading to a reduction in oxygen partial pressure. This results in a decrease in oxygen concentration at the catalyst layer interface and exacerbates concentration polarization associated with oxygen transport. Meanwhile, elevated temperatures also promote water evaporation within the membrane, reducing membrane hydration, increasing ohmic resistance, and introducing additional losses that counteract the kinetic benefits of temperature rise, ultimately leading to performance degradation. As illustrated in the Figure 10d, the stacks performance improves with increasing inlet gas pressure. However, the performance growth slows down when the pressure is increased from 100 to 120 kPa. With increasing pressure, because the water vapor in the chamber is already saturated, its partial pressure remains nearly constant. Therefore, the beneficial effect of increased oxygen partial pressure in reducing concentration overpotential gradually diminishes. It is worth noting that since the pressure difference between oxygen and cooling water must be maintained at 20 kPa. Higher inlet gas pressure requires a corresponding increase in cooling water pressure, resulting in greater energy consumption. Therefore, the optimal gas pressure should be determined by comprehensively considering the performance improvement and the additional energy consumption.

## 5 Summary and Conclusion

In this paper, a WTP was designed and fabricated to satisfy the dual requirements of passive water removal and gas tightness for dead‐ended cathode operation. A pore‐network model was proposed to interrogate capillarity‐driven transport in complex porous media. With water flux and bubble pressure being used as metrics, an optimal average pore diameter was obtained for WTP design. Static drainage PEMFC stack was then operated to exhibit superior cathode water‐management performance. The main findings are summarized as follows:

1. With a focus on pore geometry and pore connectivity, a pore network model was established. The average pore diameter of the porous plate affects its water flux and bubble pressure. Using water flux (≥27.9 µL·cm^−2^·min^−1^) and bubble pressure (≥40 kPa) as joint metrics, an appropriate average pore diameter range of 1.2–4.2 µm was identified for WTP design.
2. Enhancing the pore hydrophilicity can effectively increase the bubble pressure. For hydrophilic pores, bubble pressure is influenced by both average pore diameter and contact angle. Increasing temperature improves water flux while reducing bubble pressure due to the decline in water viscosity and surface tension.
3. The WTP with the optimal pore diameter was fabricated and assembled into the fuel cell stack. Under dead‐ended cathode operation with periodic anode purging, the stacks operated stably at 1 A·cm^−2^ for more than 30 h with a 1.92% performance decay, demonstrating both effective drainage and adequate sealing of the WTP.

Overall, the stable operation of the static drainage PEMFC is highly dependent on the design of the WTP. Understanding capillarity‐driven transport within complex pore architectures provides important guidance for the optimization of WTP design. The experiments conducted in this study do not fully replicate the environmental conditions of space or underwater applications. Therefore, further investigations are required to evaluate the adaptability of the system under more realistic operational environments. Considering the intrinsic heterogeneity of pore‐scale parameters and the perturbations introduced by subsequent fabrication, the predictive model warrants further refinement.

## Author Contributions

**Mengdi Guo**: writing – original draft, investigation, writing – review and editing, visualization, validation, methodology, conceptualization, formal analysis, software. **Diankai Qiu**: supervision, writing – review and editing, funding acquisition, resources, project administration, formal analysis. **Dong Zhu**: resources, project administration, writing – review and editing, supervision, formal analysis. **Linfa Peng**: funding acquisition, writing – review and editing, resources, supervision, project administration, formal analysis.

## Conflicts of Interest

The authors declare no conflicts of interest.

## Data Availability

The data that support the findings of this study are available from the corresponding author upon reasonable request.
